# Treatment of idiopathic internal jugular vein thrombosis in a healthy woman with enoxaparin and rivaroxiban: Case report and literature narrative review

**DOI:** 10.1016/j.amsu.2022.104526

**Published:** 2022-10-06

**Authors:** Mhd Kutaiba Albuni, Bisher Sawaf, Elias Battikh, Mohammed Nasser, Fahmi Khan

**Affiliations:** a Faculty of Medicine, Damascus University, Damsscus, Syria; b Faculty of Medicine, Syrian Private University, Damascus, Syria; c Internal Medicine Department, Hamad Medical Corporation, Doha, Qatar

**Keywords:** Venous thrombosis, Internal jugular vein, Idiopathic thrombosis, Case report

## Abstract

**Introduction:**

Venous thrombosis is a medical condition that occurs when a blood clot forms in a vein. These clots usually develop in the lower leg, thigh, or pelvis but can also occur in the arm. It is essential to know about Venous thrombosis because it can happen to anybody and cause severe illness and disability. Fortunately, if the diagnosis is early, the outcomes will be excellent. However, idiopathic or spontaneous internal jugular vein thrombosis is a rare but potentially fatal condition. Method: here, we presented a rare case of Internal jugular vein thrombosis (IJVT) and reviewed the literature on cases of IJVT to describe clinical features, associated risk factors, possible complications, ways of investigations, and outcomes.

**Results:**

Among 57 cases (56 in the literature plus our case), 25 patients out of 57 had a chief complaint of neck swelling, and only five complained of neck pain; on the other hand, four patients were asymptomatic. Thirty-five patients had a risk factor of developing thrombosis, 19 patients had a malignancy, and 22 did not have an obvious risk factor. To diagnose IJVT, ultrasound alone was used in 11 patients, Ct alone was used in 13 patients, and a combination of CT and ultrasound was used in 21 patients. Conclusion: IJVT thrombosis is a rare condition, but its diagnosis requires reasonable radiological and laboratory investigations; early treatment is warranted to avoid fetal complications.

## Highlights

• Thrombosis is the term for the growth of a blood clot within a blood vessel, which may happen in either veins or arteries.
•Following a proper diagnosis, this condition is regarded as an urgent disease that has to be treated right once.
•The disorder known as idiopathic internal jugular vein thrombosis is very uncommon.
•Radiological and laboratory tests should rule out any other causes of type II jugular vein thrombosis.

## 1 Introduction

**Fig. 1:**
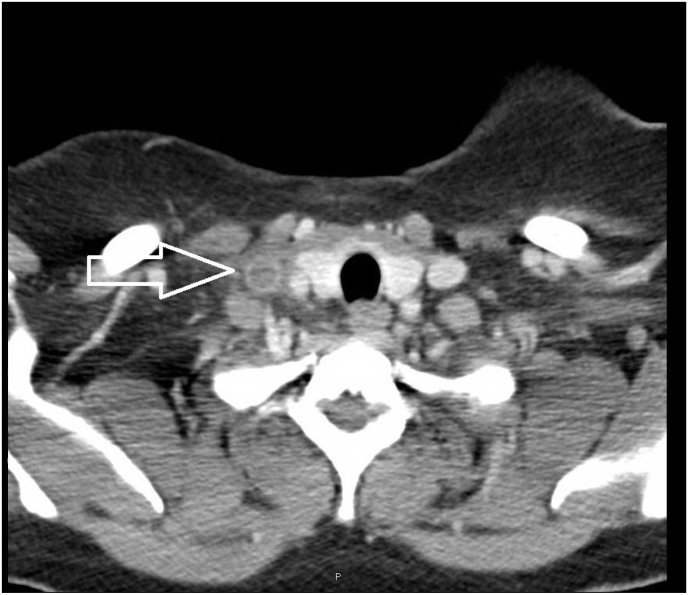
Contrast-enhanced computed tomography showing thrombosis in the right internal jugular vein.

Thrombosis is the formation of a blood clot inside a blood vessel, which may occur in veins (venous thrombosis) or arteries (arterial thrombosis). Venous thrombosis is an intravascular condition resulting from the alteration of blood constituents (hypercoagulable states), alterations in blood flow, and vascular endothelial injuries. Internal jugular vein thrombosis (IJVT) is an unusual case of vascular disease of the upper limb veins that could result in multiple complications if left untreated. IJVT can be subdivided into primary and secondary. Primary IJVT is when thrombosis happens to someone without known risk factors. Most patients present with painful erythematous neck swelling and headache. The duplex/Doppler ultrasound is the best initial test to define the extent of the thrombosis [1,2]. Ultrasound has the crucial advantage of providing a bedside diagnosis with high sensitivity and specificity and may achieve a superior resolution to computed tomography in superficial areas [3]. Anticoagulation is the treatment of choice for patients with internal jugular vein thrombosis [4]. Anticoagulant therapy following a diagnosis of IJV thrombosis prevents severe complications such as pulmonary embolism. Here, we present a rare case of idiopathic internal jugular vein thrombosis treated with enoxaparin. We followed up on the patient's condition for six months by prescribing oral rivaroxaban, Where the patient's condition improved. To develop a better understanding of the risk factors, clinical manifestations, the diagnostics used, ways of management, and complications, we further reviewed 57 reported cases of IJVT This case with narrative review was written depending on checklist guidelines for reporting case reports [5] (see Fig. 1).

## 2 Methods

### 2.1 Data sources

We reviewed the English-language literature and checked the relevant references from 1970 to 2020 to identify all known cases of Thrombosis of the Right Internal Jugular Vein using multiple sources; MEDLINE, PubMed, search engine Google and EMBASE using the terms jugular vein thrombosis, Internal jugular vein thrombosis, idiopathic Internal jugular thrombosis. Manuscripts published in English and Spanish were reviewed and relevant references were checked, and authors were contacted where possible. In addition, we also present a new case report.

### 2.2 Inclusion & exclusion criteria

All cases with a diagnosis of internal jugular vein thrombosis (with or without clinical signs), were included in this report [[6], [7], [8], [9], [10], [11], [12], [13], [14], [15], [16], [17], [18], [19], [20], [21], [22], [23], [24], [25], [26], [27], [28], [29], [30], [31], [32], [33], [34], [35], [36], [37], [38], [39], [40], [41]]. We excluded cases if the abstracts had deficient clinical data.

### 2.3 Data extraction

For all reports of Internal jugular vein thrombosis, the information extracted included, if available: patient's age and sex, clinical and investigation tools used to confirm the diagnosis, another site of thrombosis, associated medical conditions, complications as well as the outcome.

### 2.4 Results

Fifty-seven cases that meet the criteria were included in the review. Table 1 lists, in addition to our case, all patients’ characteristics in cases of IJV thrombosis. The patients were aged from 16 to 88 (50.2 +- 20 standard deviation of years). Twenty-four were males (30–78) and 33 were females (16–88). With regards to possible risk factors for thrombosis, 19 patients were diagnosed with a malignancy, two patients had the antiphospholipid syndrome, two patients had the nephrotic syndrome, one patient had homozygous factor v Leiden mutation and activated protein C resistance, one patient had Homozygous MTHFR mutation and protein C deficiency, one patient had protein S deficiency, one patient had septic pneumonia, one patient has a liver transplant, one patient was a Nitrous oxide abuser, one patient had ovarian hyperstimulation syndrome, one patient had hypothyroidism, one patient had a substernal goiter, and one patient had a laryngeal surgery. However, 22 patients did not have any associated factors to IJV thrombosis.

**Table 1:** Data analysis.

Variable	Cases of Internal jugular vein thrombosis
Sex, n(%)	
male	24 (42.11%)
female	33 (57.89%)
Mean age, years (range)	50.2 (16–88)
Investigations used, n (%)	
US	11 (19.29%)
CT	13 (22.08%)
MRI	2 (3.50%)
US + CT	21 (36.84%)
US + CT + MRI	2 (3.50%)
CT + FDG PET	1 (1.75%)
**US + MRI**	2 (3.50%)
**MRI + Angiography**	1 (1.75%)
**Veinography + US**	1 (1.75%)
**Not specified**	2 (3.50%)
CT + MRI	1 (1.75%)
Location of IJV thrombosis n, (%)	23 (40.35%)
Left IJV	23 (40.35%)
Right IJV	7 (12.28%)
Bilateral IJV	4 (7.01%)
Not specified	6 (10.52%)
Involvement of other veins n, (%)	2 (3.50$)
Subclavian vein	1 (1.75%)
Subclavian and brachiocephalic	1 (1.75%)
Subclavian and axillary	1 (1.75%)
Subclavian, axillary, and humeral	1 (1.75%)
External jugular	1 (1.75%)
External Jugular and brachiocephalic	1 (1.75%)
Sigmoid sinus	42 (73.68%)
Transverses and sigmoid sinus	19 (33.33%)
None	2 (3.50%)
Associated Factors n, (%) Malignancy	1 (1.75%)
Antiphospholipid syndrome	1 (1.75%)
Homozygous factor V Leide**n Mutation, activated factor C** resistance and Dengue fever	1 (1.75%)
Homozygous MTHFR mutation and protein C deficiency Protein S deficiency	1 (1.75%)
Septic Pneumonia and HIT	1 (1.75%)
**Liver Transplant** Ovarian hyperstimulation syndrome	2 (3.50%)
Nephrotic Syndrome	1 (1.75%)
Nitrous Oxide abuse	1 (1.75%)
Hypothyroidism	1 (1.75%)
Substernal Goiter	1 (1.75%)
Laryngeal surgery	1 (1.75%)
**None**	**22 (38.59%)**

Clinical Presentation	Case of IJV thrombosis
Neck Swelling	25 (43.85%)
Asymptomatic	4 (7.05%)
Neck Pain	5 (8.77%)
Swelling of the supraclavicular fossa	1 (1.75%)
Chest pain	2 (3.50%)
Facial and upper limb swelling	2 (3.50%)
Swelling of the parotid gland	1 (1.75%)
Fever and dyspnea	2 (3.50%)
Dizziness	1 (1.75%)
Hoarseness	1 (1.75%)
Confusion and fall	1 (1.75%)
Septic shock and encephalopathy	1 (1.75%)
Exacerbation of COPD	1 (1.75%)

Complication	Cases of IJV thrombosis
No complication	48 (84.21%)
Death	2 (3.50%)
Peripheral facial palsy	1 (1.75%)
Pulmonary embolism	2 (3.50%)
Pulmonary HTN	1 (1.75%)
Not specified	3 (5.26%)

Diagnostic modalities were different among the cases: Ultrasound was used in 11 cases, computed tomography (CT) scan was used in 13 cases, but the combination of CT and ultrasound was used in 21 cases, and Magnetic resonance imaging (MRI) was used in 2 cases, the combination of MRI and US was used in two cases, the combination of CT and MRI was used in one case, the combination of US and MRI was used in two cases. Venography and US were used in one case, and MRI with angiography was used in one case.

Side of IJV thrombosis was distributed equally, with 23 cases involving left IJV and 23 cases right IJV, but 7 cases involved both sides. The involvement of other veins was reported in 13 cases. In 6 cases subclavian vein was involved; in two cases, subclavian and brachiocephalic were involved. One case involved subclavian and axillary veins, and one case involved subclavian, axillary, and humeral veins. One case involved the external jugular vein, one case external and brachiocephalic, one case involved sigmoid sinus, and one involved transverse and sigmoid sinus.

The clinical manifestation of IJV thrombosis showed a range of variety in most cases, 25 presented with neck swelling, five patients with neck pain, and 4 cases were asymptomatic. The other cases present with supraclavicular fossa swelling, facial and upper limb swelling, fever and dyspnea, dizziness, confusion and fall, chest pain, and parotid gland swelling.

In most of the cases, 48 were treated without complications. However, two ended up with death; two patients had a pulmonary embolism, one had pulmonary hypertension, and one had peripheral facial nerve palsy.

## 3 Case report

A 27-year-old Kenyan female with no history of previous illness presented to the emergency department with a 7-day history of neck pain and swelling. Her medical and family histories are unremarkable. She denied taking oral contraceptives or IV drugs. Also, she banned fever, loss of weight, previous abortions, and a history of deep vein thrombosis. On examination, she looked well with stable vital signs. A neck examination showed right-sided neck swelling, which was tender upon palpation. Examination of the ipsilateral and contralateral arms, as well as the other systems, was routine. Urgent neck ultrasound doppler showed right internal jugular vein thrombosis. Her CBC, blood chemistry, liver function test, vitamin B12 level, and coagulation profile were within normal limits. Chest radiography did not reveal any cervical rib, and a contrast-enhanced computed tomography (CT) pulmonary angiography was unremarkable. Thrombophilia workups including ANA, antiphospholipid Ig G/Ig M, protein C, protein S, antithrombin III activity, homocysteine, and fibrinogen level were requested, and the patient was admitted to the medical ward, and subcutaneous enoxaparin was initiated.

In the ward, enoxaparin was stopped, and rivaroxaban was started. Thrombophilia workups return negative, ruling out secondary causes of thrombosis and supporting the diagnosis of idiopathic thrombosis of the right internal jugular vein. CT abdomen and head were also unremarkable. The patient was discharged on the 5th day of admission in good condition to continue rivaroxaban for three months.

### 3.1 Discussion

Internal jugular vein thrombosis refers to an intraluminal thrombus occurring anywhere from the intracranial internal jugular vein to the junction of the internal jugular and the subclavian vein to form the brachiocephalic vein. The internal jugular vein is an uncommon site of spontaneous venous thrombosis. It is an underdiagnosed condition that may occur as a complication of head and neck infections, surgery, central venous access, local malignancy, polycythemia, hyperhomocysteinemia, neck massage, and intravenous (IV) drug abuse. It is also reported to occur spontaneously as a rare case [42]. Internal jugular vein thrombosis tended to occur more often in women. Most patients are asymptomatic on presentation; however, some do present with the classic signs of DVT which include erythema, swelling, and tenderness [43]. Internal jugular thrombosis itself can have serious potentially life-threatening complications, including systemic sepsis, chylothorax, papilledema, airway edema, and pulmonary embolism (PE). Internal jugular thrombosis is usually divided into primary thrombosis (i.e. idiopathic thrombosis and thrombosis associated with the thoracic outlet syndrome or effort) and secondary thrombosis [44]. In cases of unprovoked IJV thrombosis, oncological disease and thrombophilia should be screened for since they are more common than in cases of lower-body thrombosis. patients with idiopathic internal jugular vein thrombosis may undergo systematically biochemical tests, i.e. antiphospholipid, anticardiolipin, and anti-b2-GPI antibodies as well as lupus anticoagulant, in order to depict underlying primary antiphospholipid antibody syndrome. D-dimer should be done before proceeding to CT imaging whenever thrombosis is suspected, as it has a high negative predictive value. However, a negative D-dimer test cannot rule out thrombosis in patients with suggestive symptoms and predisposing conditions [45]. Performance of imaging to diagnose IJV is unknown. The majority of reports used ultrasonography often completed with CT. Duplex ultrasonography is the diagnostic modality of choice as it is non-invasive and inexpensive. It has a sensitivity ranging from 78% to 100% and a specificity of 82%–100% [46]. The goal of treatment should be the resolution of the symptoms and prevention of recurrent episodes and further complications. The best therapy for non-cancer patients is high-dose direct oral anticoagulants. Treatment duration is at least 3 months, with possible long-term anticoagulation depending on the cause of thrombosis [47]. Compared to other articles, about 20 cases of primary internal jugular thrombosis have been reported in the medical literature as case reports. The most important thing that can be noted when adding such a rare case is the accurate evaluation of the patient who presents a complaint of swelling or tenderness in the neck without the presence of previous diseases. Our patient is a 27-year-old female who presented with swelling of the right side of her neck with tenderness. An ultrasonographic scan showed thrombosis of the right jugular vein. Clinical tests, including X-ray, CT scan and abdominal ultrasonography, Serological analysis of coagulation, were negative. The patient was heparinized immediately using enoxaparin and then underwent rivaroxaban anticoagulation therapy for 3 months. One year later the patient was in good clinical condition.

### 3.2 Conclusion

Idiopathic internal jugular vein thrombosis is a rare condition. All other causes should be excluded radiologically and by laboratory tests related to type II jugular vein thrombosis. Idiopathic internal jugular vein thrombosis has potentially fatal complications. Therefore, a physician should provide primary care when they suspect the presence of jugular vein thrombosis or when there is neck swelling with tenderness on palpation.

## Ethical approval

N/A.

## Sources of funding

N/a.

## Consent

Written informed consent was obtained from the patient for publication of these two case reports and accompanying images. A copy of the written consent is available for review by the Editor-in-Chief of this journal.

## Author contribution

MA: Manuscript writing, Data analysis, and approval of the final manuscript
BS: manuscript writing and editing, literature review, review and approval of the final manuscript
EB: manuscript writing, approval of the final manuscript
MN: manuscript writing
FK: Case selection, case identification and conceptualization, literature review, manuscript writing, obtaining informed written consent, prescribing medicine, clinical follow up, and mentor

## Registration of research studies

Not applicable.

## Guarantor

Fahmi Khan.

## Declaration of competing interest

All authors declare no conflict of interest.
